# First-principle study on the stability of Cd passivates in soil

**DOI:** 10.1038/s41598-023-31460-8

**Published:** 2023-03-14

**Authors:** Jianglong Shen, Juan Li, Zhongan Mao, Yang Zhang

**Affiliations:** 1 Key Laboratory of Degraded and Unused Land Consolidation Engineering, Ministry of Natural Resources, Xi’an, 710075 China; 2 Institute of Land Engineering and Technology, Shaanxi Provincial Land Engineering Construction Group Co., Ltd., Xi’an, 710075 China; 3 Shaanxi Provincial Land Engineering Construction Group Co., Ltd., Xi’an, 710075 China; 4 Shaanxi Provincial Land Consolidation Engineering Technology Research Center, Xi’an, 710075 China

**Keywords:** Biogeochemistry, Environmental sciences, Chemistry, Engineering, Materials science, Mathematics and computing

## Abstract

The stable existence of heavy metals in soil under natural conditions is the core issue in heavy metal pollution solidification and remediation technology. However, the existing research is limited to soil passivation tests of different materials or biochar adsorption tests and cannot reveal the internal mechanism of functional groups of different compounds in soil passivation. This paper takes the common heavy metal ion Cd^2+^ as an example to analyze the stability of the combination of heavy metal ions and common ion groups in soil. The stability and existing form of Cd are analyzed by using first-principle calculations, and the free energy, band structure, and partial density of states of CdCO_3_, CdSO_4_, CdCl_2_, and CdSiO_3_ are computed. The stability of Cd binding to common anions in soil is determined. Results show the descending order of structural stability of cadmium compounds is CdSiO_3_, CdSO_4_, CdCO_3_, and CdCl_2_. SO_4_^2−^ and SiO_3_^2−^ can be used as preferred functional groups for cadmium pollution passivation. Anhydrous sodium sulfate and sodium silicate are promising passivators.

## Introduction

With the development of society, the degree of industrialization is increasing. The industrial waste generated by industrial production activities as the source of heavy metal pollution is usually not properly treated^1–3^. In the long run, the pollution sources are increasing, and more heavy metals enter the soil through various ways and then continue to accumulate in the soil to form pollution^4–8^. Soil plays a fundamental role in food safety, and the adverse effects of contaminants like heavy metal (loid)s on crop quality have threatened human health^9–17^. The theoretical optimal scheme for remediation of agricultural nonpoint source heavy metal pollution is to add amendments in contaminated soil, change the heavy metal elements from bioavailable state to residual state, limit the migration of heavy metal ions, and achieve in-situ remediation. To improve research and development, knowing the physical and chemical properties of heavy metal pollutants is of great importance. Understanding the solidification, adsorption, and precipitation of such pollutant heavy metals under normal temperature and pressure is the key research content. At this stage, the nature of soil Cd pollution is mainly determined through experiments, such as infrared scanning, Raman laser spectroscopy, and X-ray diffraction^18–21^. Phosphoric acid and sulfur compounds can be used as stabilizers of cadmium. However, the experimental observation results cannot explain the theoretical problems, and one observation cannot ensure that it conforms to the laws of physics and chemistry. In addition, the precipitation conditions of cadmium-containing compounds are different from the natural state of soil chemistry^22,23^. The stability of cadmium-containing compounds cannot be fundamentally solved by traditional chemical theories. The above problems can be solved by using quantum geochemical tools to calculate the stability of the combination of CO_3_^2−^, SO_4_^2−^, Cl^−^, SiO_3_^2−^ plasma, and Cd^2+^. At present, reports on the theoretical study of the energy stability of various cadmium compounds are few.

First-principle calculation has been widely used in many scientific research directions, such as the simulation of unknown material properties in materials science, such as the design and synthesis of functional photoelectric materials by calculating the photoelectric properties of materials. In geology, it is used to simulate the structure and composition^10,24–28^. In thermodynamics, the simulated formation and configuration of minerals are studied. The research on passivating agent for in-situ remediation of heavy metal pollution is still in the research stage of what kind of substance is used as passivating agent and its passivating effect^29^. Few studies have applied the first-principle calculation in the calculation of heavy metal passivation. Common acid ions, such as chloride ion, carbonate ion, silicate ion, and sulfate ion, are introduced into the calculation of cadmium containing system by using first-principle calculation. In this way, the stability of the combination of functional groups and Cd^2+^ can be better understood to design more suitable soil remediation passivators.

## Calculation principle and method

According to the principle of interaction between nucleus and electron and its basic motion law, the principle of quantum mechanics is applied, and the Schrodinger equation is solved directly after some approximate processing algorithm, called first-principle calculations. First-principle calculations include two major categories, Hartree–Fock self-consistent field calculations based on ab initio and density functional theory (DFT) calculations^30^. The first-principle calculation using Vienna Ab initio Simulation Package (VASP)^31,32^ software in this paper is based on DFT. At normal temperature and pressure, Gibbs free energy plays a decisive role in material stability. The Gibbs free energy calculation formula is G = H − TS, H = U + PV, where G is the Gibbs free energy, H is the enthalpy, T is the Fahrenheit temperature, S is the entropy, U is the internal energy of the system, P is the pressure, and V is the volume. H, T, and S are state functions. G is a state function, representing the properties of a particular state. Given that the entropy value has difficulty determining the exact value, the Fahrenheit temperature is set as absolute zero, the crystal structure from absolute zero to normal temperature and pressure has not been deformed, and the properties are consistent. Hence, extrapolating the properties calculated from the free energy under absolute zero to those under normal temperature and pressure is reasonable. Under stable conditions, substances react in the direction of lower Gibbs free energy to form substances with lower Gibbs free energy value, which can be used to judge the stability and reaction direction of heavy metal compounds^33,34^.

In this paper, crystal coefficient files in the crystal library are needed to establish the corresponding crystals, process them with CATSEP in Materials Studio, and then place them into VASP package for first-principle calculation. The projection method is projection augmented wave method, and PAW_PBE is selected as a pseudopotential of projected augmented wave, which functions as PBE exchange key energy functional. The electron orbit of cadmium is corrected by GGA + U treatment of 3d electron orbit and then calculated accordingly. Parameters in the calculation are set as follows: PREC = accurate, and EDIFF = 1.E−05 conversion unit is set as eV, that is, the convergence point energy limit is 10^−5^, and the corresponding convergence energy limit between atoms is 10^−2^ eV/nm. In the optimization, the optimized NSW = 200 is selected, that is, the number of motion steps in the nucleus is 200, and the subsequent self-consistent motion in the nucleus keeps the nucleus stationary, that is, NSW = 0. The calculation of each system is carried out after the convergence test, in which the truncation energy and the parameter setting of Brillouin zone location point (K point) adopt the corresponding value according to the different calculation systems, and the selection of K point adopts the Monkhorst Pack method. The plane wave truncation energy is optimized by using the equal difference number sequence of 200, 250, 300, 350, 400, 450, 500, 550, and 600 eV to determine that 500 eV is the most appropriate truncation energy because the structural optimization part and the energy calculation part have different requirements for accuracy.

The expansion width of heavy metal cadmium in the calculation parameter setting is selected as SIGMA = 0.05. In the K-point selection, considering accuracy and calculation cost, the optimization should be as large as possible when the calculation accuracy allows. When calculating the density of states, the K-point value should be relatively reduced. The highly symmetric K-point distribution is adopted in the calculation of energy band.

## Results and discussion

### Selection and optimization of crystal structure

**Figure 1:**
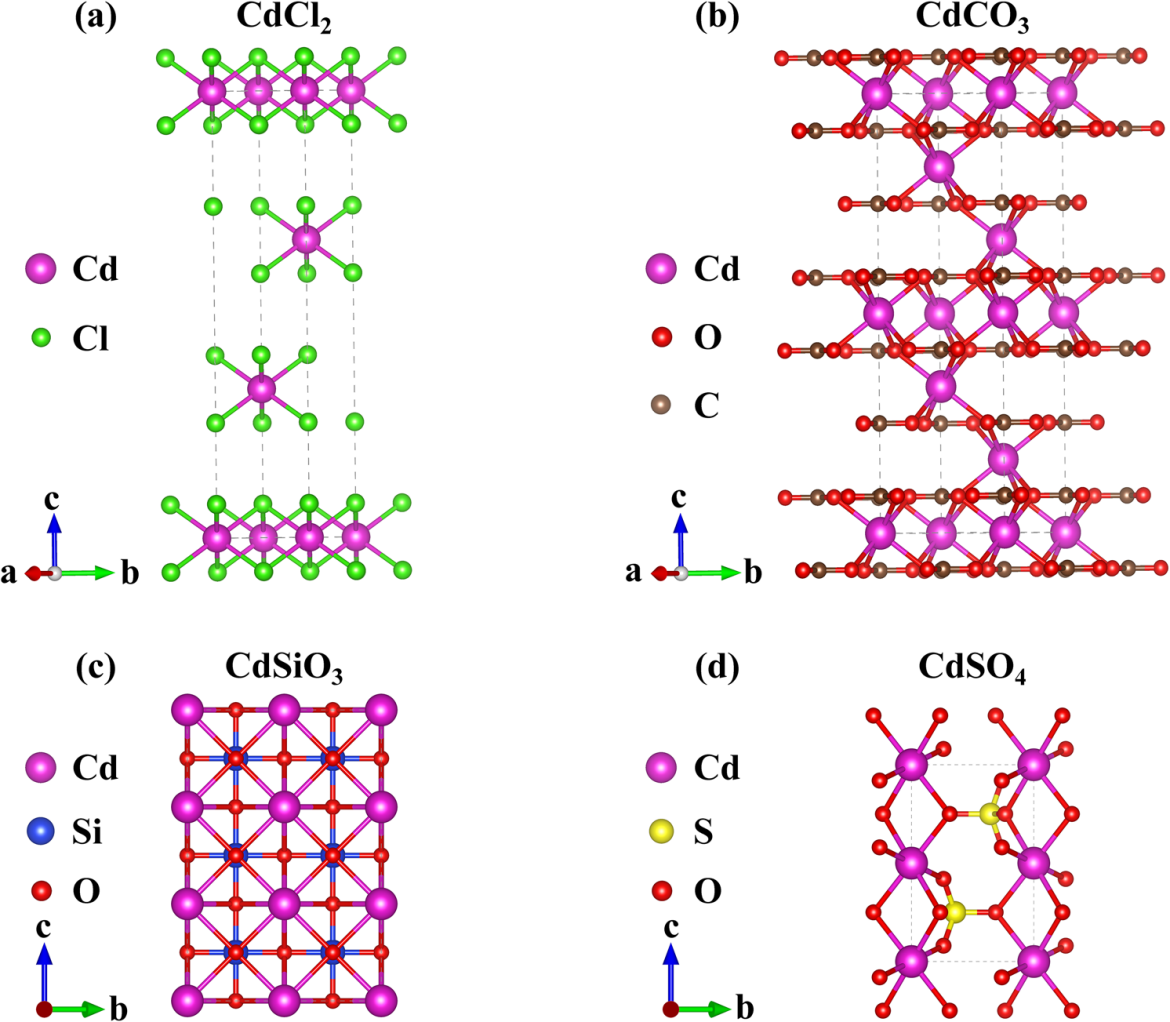
Cell structure of CdCl_2_, CdCO_3_, CdSiO_3_, and CdSO_4_.

After comprehensively considering factors such as the difficulty of obtaining cadmium compound groups, purity, and process level, the type of cadmium-containing crystals used for calculation was selected, which provides reference for the selection of passivating agents in the later stage. The anion group is chloride ion, carbonate, silicate, and sulfate, and the chemical formulas are CdCl_2_, CdCO_3_, CdSiO_3_, and CdSO_4_, respectively. The corresponding crystal structures of the above compounds were obtained from the Crystallography Open Database in Fig. 1.

Table 1 shows the cell structure parameters of four cadmium compounds after optimization. The unit cell parameters optimized by VASP calculation are very close to the ideal experimental values, and the change ratio is within 0.2%, which further indicates the theoretical calculation by VASP is the same as the experimental results under ideal conditions. The deviation between the bond length and bond angle in the unit cell and the ideal experimental value after VASP optimization is within 0.8%, which indicates VASP does not substantially change the original structure of the unit cell when calculating these units, and cadmium and corresponding ionic groups in the unit cell can maintain the original physical and chemical properties.

**Table 1:** Cell parameters of CdCl_2_, CdCO_3_, CdSiO_3_, and CdSO_4_.

Model	Lattice constant (Å)			Shaft angle			Volume (Å^3^)
	a	b	c	α	β	γ	
CdCl_2_	3.9259	3.9259	18.9468	90	90	120	252.8943
CdCO_3_	4.9204	4.9204	16.298	90	90	120	341.7164
CdSiO_3_	7.2398	7.2398	10.8598	90	90	90	569.2174
CdSO_4_	4.9978	4.9978	7.3134	90	90	90	166.0376

### Free energy

The energy system of CdCl_2_, CdCO_3_, CdSiO_3_, and CdSO_4_ at room temperature and pressure is calculated by the first-principle software VASP, as shown in Table 2. The ascending order of Gibbs free energy of the four cadmium containing systems from is CdSiO_3_, CdSO_4_, CdCO_3_, and CdCl_2_. The relative stability of these systems is inversely related to their corresponding Gibbs free energy ordering. The predicted descending order of the relative structural stability of four simple cadmium compounds is CdSiO_3_, CdSO_4_, CdCO_3_, and CdCl_2_.

**Table 2:** Energy parameters of CdCl_2_, CdCO_3_, CdSiO_3_ and CdSO_4_.

System	Fermi level (eV)	Free energy (J/g atom)
CdCl_2_	− 0.368	− 7.8643124
CdCO_3_	1.4326	− 63.875465
CdSiO_3_	5.4327	387.048856
CdSO_4_	1.7624	− 67.875434

### Band structure and partial density of states

**Figure 2:**
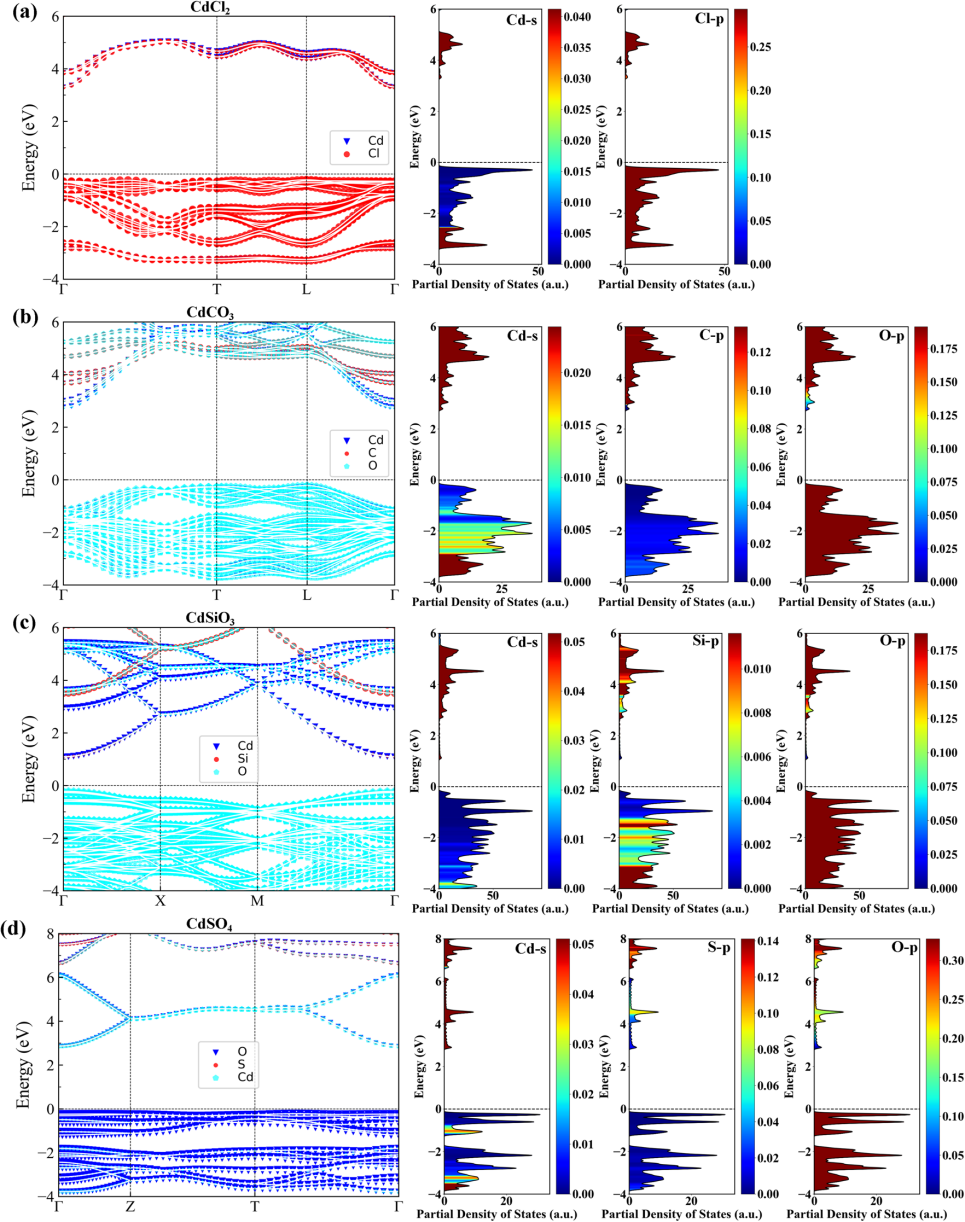
Band structure (left) and partial density of states (PDOS) (right) of four systems: (**a**) CdCl_2_, (**b**) CdCO_3_, (**c**) CdSiO_3_, and (**d**) CdSO_4_ (Note: 0 eV represents its corresponding Fermi energy level, where the abscissa Γ Gamma point, the center of Brillouin zone).

By calculating the band values of the above systems, the band structures are derived, and more detailed structural information of these systems is obtained to understand the electrical properties, crystal configurations, and chemical bond types of these systems deeply, and determine the functional groups polluted by cadmium passivation better^35–40^. Figure 2 shows the Fermi energy level (Efermi) is taken as the energy zero. The band gap widths of CdCl_2_, CdCO_3_, CdSiO_3_, and CdSO_4_ are 3.53, 2.98, 1.32, and 3.09 eV, respectively. These four systems are semiconductors, but their conductivity is weak. Figure 2 shows the top of the valence band of the four systems, CdCl_2_, CdCO_3_, CdSiO_3_, and CdSO_4_, is closer to its corresponding Fermi energy level in the system (the position of 0 eV in the figure), and the conduction band of these systems is more volatile. These systems are p-type semiconductors^41–44^. In addition, the crystal structure of these systems is not filled with electrons, and attracting electrons to fill the crystal holes is easier when the properties change. The energy band of this series of systems is narrow due to the relatively dense electrons in the energy band position in the crystal structure, which is caused by the overlap of some hybrid orbitals. It has more effective electrons and higher relative mass. These systems fluctuate greatly in the direction from the center of the Brillouin zone to the center of the quadrilateral surface, so more attention should be paid to the selection and manufacture of passivation that change their structural properties.

Partial density of states (PDOS) is another way to present the crystal structure. The combination of PDOS and energy band structure map in Fig. 2 can better reflect its crystal structure properties. The size of PDOS can replicate the strong degree of the combination of cations and anions in the system, thus inferring the relative stability order of these systems. In addition, PDOS can directly reflect the influence of different anion groups on the electronic density of the whole system^45–50^. Figure 2 shows the PDOS of CdCl_2_, CdCO_3_, CdSiO_3_, and CdSO_4_ systems have local sharpening peaks in the conduction band and the valence band. The electrons in these areas are denser than those in other locations in the crystal, and the presented aggregation rule is mutually corroborated by the narrower energy band in the corresponding location displayed in the energy band structure^51^. The dispersion of the valence band in the CdSO_4_ system is higher than that of CdSiO_3_, CdCO_3_, and CdCl_2_, indicating the binding force of Cd^2+^ and SO_4_^2−^ is stronger.

In the CdCl_2_ system, the contribution of the s orbital of Cd to the system is mainly reflected in the bottom of the conduction band, and the main contribution of the p orbital of Cl to the system is reflected in the valence band. In the CdCO_3_ system, the main contribution of the s orbit of Cd to the system is reflected in the bottom of the conduction band, the main contribution of the p orbit of C to the system is reflected in the bottom of the conduction band, and the main contribution of the p orbit of O to the system is reflected in the valence band. In the CdSiO_3_ system, the contribution of the s orbital of Cd to the system is mainly reflected in the bottom of the conduction band, the contribution of the p orbital of Si to the system is mainly reflected in the conduction band, and the contribution of the p orbital of O to the system is reflected in the valence band. In the CdSO_4_ system, the contribution of the s orbital of Cd to the system is mainly reflected in the conduction band, and the contribution of the p orbital of O to the system is mainly reflected in the valence band. According to the calculated original PDOS values, the electron density in these four systems is high in the s-orbital contribution rate and low in the p-orbital contribution rate. Table 3 describes the density of state percentage and the density of state ratio of the anion and cation groups of various compounds containing cadmium in the system. Among the selected series, SO_4_^2−^ accounts for the highest proportion of anions in the compound system containing cadmium, which plays a decisive role in stability, and SiO3^2−^ is also better than Cl^−^. Thus, SO_4_^2−^ and SiO_3_^2−^ may be the preferred functional groups for cadmium ion passivation.

**Table 3:** Proportion of groups in CdCl_2_, CdCO_3_, CdSiO_3_ and CdSO_4_.

System	Anion group ratio (%)	Cation group ratio (%)	Anion/cation
CdCl_2_	20.43	27.14	0.75
CdCO_3_	47.83	27.52	1.73
CdSiO_3_	48.31	26.98	1.79
CdSO_4_	51.32	19.38	2.64

### Test application

According to first-principle calculation, the best functional groups for controlling cadmium pollution were obtained. When selecting passivating agent in practical application, the mature process and easy release of SiO_3_^2−^ and SO_4_^2−^ should be considered first. Therefore, anhydrous sodium sulfate (NaSO_4_) and sodium silicate (Na_2_SiO_3_·9H_2_O) with mature technology and easy to release cation exchange and anionic functional groups should be prioritized as passivating agents. Although the production of these samples is mature, they face the problem of high price when used in large quantities, so finding low-cost, efficient substitutes is necessary. The improvement method was to select low-cost industrial byproduct desulfurized gypsum (the main component is CaSO_4_·2H_2_O, the same as natural gypsum, content, 93%) as passivating agent. To verify the control effect of the above-mentioned three passivating agents on cadmium pollution, the natural soil of Guanzhong was used as raw material to prepare cadmium-contaminated soil by adding compounds that can release Cd^2+^. Cd(II) standard liquid, sodium dihydrogen phosphate (NaH_2_PO_4_), sodium sulfate (Na_2_SO_4_), desulfuration gypsum (CaSO_4_·2H_2_O, content ≥ 93%), and diethylenetriaminepentaacetic acid were purchased from Shanghai Chemical Reagents Company (China), Cd(II) standard liquid was guaranteed reagent, and others were analytical reagents. Thrice the cadmium pollution control value (500 mg/kg) of CdCl_2_ was added to the pollution-free soil. CdCl_2_ was dissolved in water and evenly poured into the pollution-free original soil. To shorten the duration of the experiment, the soil added with pollutants was placed in a constant temperature and humidity incubator for aging treatment, and the aging duration was 40 days. The initial mass fraction of Cd^2+^ in the self-made contaminated soil was 1432.5 mg/kg. Sodium dihydrogen phosphate, anhydrous sodium sulfate, and desulfurization gypsum (93% purity) of 2% of the soil mass to be treated were added to the self-made contaminated soil. The soil was stirred once a day, and the mass fraction of available cadmium in soil was measured after 3, 7, and 15 days of treatment. The measurement results are shown in Table 4.

**Table 4:** Active mass fraction of cadmium after passivating agent addition.

Time/d	NaH_2_PO_4_	Na_2_SO_4_	CaSO_4_·2H_2_O
0	1475.2	1475.2	1475.2
3	1148.15	1187.3	1289.54
7	1043.25	1098.73	1187.49
15	896.56	935.87	1053.76

Table 4 shows the three passivators have remarkable control effects on Cd^2+^ in contaminated soil. In 15 days, the mass fraction of available cadmium can be reduced by 28.56–39.23%. Among them, anhydrous sodium sulfate has the best effect, which verifies that SO_4_^2−^ and SiO_3_^2−^ can be used as effective functional groups to control cadmium pollution.

## Conclusion and discussion

The control of soil Cd pollution has always been a hot issue in scientific research, and soil health is related to the lifeblood of human development^52,53^. Much of the existing research had experimentally focused on the remediation of contaminated soil^54–57^. These methods consider the characteristics of high activity and large specific surface area of biochar, and adsorb heavy metal elements in soil, but the characteristics of Cd compounds themselves were not fully studied. In this paper, first-principle calculations are used to study the potential of different passivators in soil for cadmium. This approach provides a new perspective on the study of environmental pollution of heavy metals. The geometry, electronic structure, state density, charge transfer, and energy conversion of different cadmium compound crystals are analyzed, and the internal mechanism of various cadmium compounds in passivation is clarified. Experimental studies show this method is feasible.

According to first-principle calculation, the Gibbs free energy of the four cadmium-containing systems is in the ascending order of CdSiO_3_, CdSO_4_, CdCO_3_, and CdCl_2_, and the structural stability of cadmium compounds is in the descending order of CdSiO_3_, CdSO_4_, CdCO_3_, and CdCl_2_.

Based on the discussion of energy band structure and PDOS, CdCO_3_, CdSO_4_, CdCl_2_, and CdSiO_3_ are all p-type semiconductors. The crystal structures of these systems are not filled with electrons, and they are more likely to attract electrons to fill the crystal cavities when the properties change. However, the n-type semiconductor of CdSiO_3_ system shows metallicity. The electrons in the lattice are more active and can migrate and conduct in the lattice. Losing the electrons in the crystal is easier when the properties change.

In the cadmium-containing system, CO_3_^2−^, SO_4_^2−^, Cl^−^, SiO_3_^2−^, and other ionic groups are analyzed, and SO_4_^2−^ and SiO_3_^2−^ can be used as the preferred functional groups for cadmium-contaminated passivation. Anhydrous sodium sulfate and sodium silicate are effective passivating agents. If cost and large area promotion are considered, low-cost desulfurization gypsum is preferred.

## Data Availability

The datasets generated and analysed during the current study are not publicly available due this experiment was a collaborative effort, the trial data does not belong to me alone but are available from the corresponding author on reasonable request.
